# Separating positional noise from neutral alignment in multicomponent statistical shape models

**DOI:** 10.1016/j.bonr.2020.100243

**Published:** 2020-01-11

**Authors:** E.A. Audenaert, J. Van den Eynde, D.F. de Almeida, G. Steenackers, D. Vandermeulen, P. Claes

**Affiliations:** a Department of Orthopedic Surgery and Traumatology, Ghent University Hospital, Corneel Heymanslaan 10, 9000 Ghent, Belgium; b Department of Human Structure and Repair, Ghent University, Corneel Heymanslaan 10, 9000 Ghent, Belgium; c Department of Trauma and Orthopedics, Addenbrooke's Hospital, Cambridge University Hospitals NHS Foundation Trust, Hills Road, Cambridge CB2 0QQ, UK; d Department of Electromechanics, Op3Mech research group, University of Antwerp, Groenenborgerlaan 171, 2020 Antwerp, Belgium; e Centre for Rapid and Sustainable Product Development, Polytechnic Institute of Leiria, Portugal; f Department of Electrical Engineering, ESAT/PSI, KU Leuven, Kasteelpark Arenberg 10, box 2441, 3001 Leuven, Belgium; g Department of Human Genetics, KU Leuven, Herestraat 49, box 602, 3000 Leuven, Belgium; h Murdoch Children's Research Institute, Royal Children's Hospital, Flemington Rd, Parkville 3052, Melbourne, Victoria, Australia; i Department of Biomedical Engineering, University of Oxford, Old Road Campus Research Building, Headington, Oxford OX3 7DQ, United Kingdom; j Medical Imaging Research Center, MIRC, University Hospitals Leuven, Herestraat 49 - 7003, 3000 Leuven, Belgium

**Keywords:** Image analysis, Sex dimorphism, Geometric morphometrics, Multivariate regression, Anatomy

## Abstract

Given sufficient training samples, statistical shape models can provide detailed population representations for use in anthropological and computational genetic studies, injury biomechanics, musculoskeletal disease models or implant design optimization. While the technique has become extremely popular for the description of isolated anatomical structures, it suffers from positional interference when applied to coupled or articulated input data. In the present manuscript we describe and validate a novel approach to extract positional noise from such coupled data. The technique was first validated and then implemented in a multicomponent model of the lower limb. The impact of noise on the model itself as well as on the description of sexual dimorphism was evaluated. The novelty of our methodology lies in the fact that no rigid transformations are calculated or imposed on the data by means of idealized joint definitions and by extension the models obtained from them.

## Highlights

• Differences in pose at the time of image acquisition adds rotational noise to articulated components in multicomponent shape models
• A PLS regression was used to learn the relation between isolated osteological structures and their alignment within the full lower limb
• Excluding positional variance, increases model compactness and improves correlations with other associated biological variables such as sex.

## 1 Introduction

Statistical shape models (SSM) have been established as a robust tool for segmentation of medical images and for the description of variation in anatomy (Almeida et al., 2016; Audenaert et al., 2019b; Cootes et al., 1994; Cootes et al., 1995). In particular, the models allow for accurate parameterization of complex data such as an individual's anatomical phenotype as well as for the realistic description of the distribution of anatomy in the population, by use of conventional multivariate statistics on dense sets of homologous landmarks representing the shape of the underlying structures (Audenaert, 2014; Heimann and Meinzer, 2009). Given sufficient training samples, they can approximate almost any naturally occurring configurations, permitting extended usage and advancements in numerous scientific areas including anthropology, evolutionary biology, forensics, implant design, anatomy, epidemiology and last but not least clinics, for the distinction of physiological versus pathological anatomical variation. (Audenaert, 2014; Baldwin et al., 2010; Henak et al., 2013).

While SSMs have become popular for the description of isolated anatomical structures, they have rarely been applied in more complex and integrated anatomies such as an entire limb or even the whole human skeleton. Usually osteological variation is analyzed in 2D, which is obviously prone to position and in particular (in plane) rotational noise(Brouwer et al., 2003). 3D analysis is more accurate in this respect, but again rotational (knee flexion, hip rotation) variation affects the measurement results and impedes in depth description of a patients neutral alignment. Different poses among scanned patients at the time of the image acquisition act as confounders which contributes to the complexity of an already challenging problem. Until now these constraint have not properly been addressed.

Previous authors have attempted to control the impact of alignment and variance in joint rotations for segmentation applications, either by defining statistics on rigid transformation models to capture differences in pose (Boisvert et al., 2008) or by explicitly modeling joint motion through idealized joint models (e.g. spherical in case of the hip or hinged for the knee joint) (Bindernagel, 2013; Kainmueller et al., 2008, Kainmueller et al., 2009). While such approaches have been shown to be effective in intentionally adding plausible variance to the data, as needed for the case of segmentation tasks, they were not designed, and to a certain extent fail, to differentiate scanned poses or degenerative malalignment from native alignment as part of the original anatomical variation. Several research domains, e.g. anthropology, evolutionary biology, however, require dense and precise anatomical information, without positional noise, for correlation studies on anatomical data to be valid (Baird et al., 2019; Lindner et al., 2015). In fact, in depth 3D anatomical studies of coupled anatomies based on geometric morphometrics are currently nonexistent. However, an improved understanding of the variation in anatomy and its association with other relevant variables such as genetic dissimilarity, is of utmost importance as it involves a fundamental determinant in musculoskeletal disease understanding, clinical decision making and directly relates to joint mechanics and wear (Allen and Pagnano, 2016; Ritter et al., 2011).

In the present manuscript we present a new approach to extract positional noise from coupled data. With coupled data we refer to two or more shape constructs, that can be modelled separately, but have an integrated, predefined positioning. Doing so we aimed to allow for improved morphological phenotyping, i.e., the description of population variance in lower limb morphology. Advantages and performance of the presented technique were evaluated by correlating the pose corrected model with the simplest and most basic of genetic variables: sex. The novelty of our methodology mainly lies in the fact that no rigid transformations are calculated or imposed on the data by means of idealized joint definitions and by extension the models obtained from them.

## 2 Methodology

### 2.1 Data acquisition and pre-processing

A total of 606 training data sets (left and right combined) were considered, originating from 303 Computed Tomography (CT) scans. Every image domain included the full lower limb anatomy, ranging from rib 12 to toes. The imaging database was constructed from living subjects receiving angio-CT scanning for vascular work-out between 2012 and 2016. CT data demonstrating metallic implants (e.g. hip and knee prosthesis) was excluded from the data-base. The participating subjects were not exposed to additional radiation for the present study. All the scans were processed on a Dell Precision M6800 Laptop (Intel Core i7 -4910MQ, 16 GB RAM, 64 bit). Each scan data set consisted of an average of 1864 slices with a pixel size between 0.575 mm to 0.975 mm. This study involving human participants was reviewed and approved by the ethics committee of the Ghent University Hospital (under reference B670201111480).

In-vivo clinical imaging of the human skeleton through CT, allows for a complete non-invasive depiction of the morphology of the osteological structures at interest for the present study. However, accurate and robust extraction of these structures from a large image database requires automated procedures. The segmentation task for the different bones included in the present study was described in detail in the work by Audenaert et al. (Audenaert et al., 2019b). Comparing automatic with manual segmentations demonstrated rooted mean squared differences ranging from 0.53 to 0.76 mm with the largest differences found in the pelvic bones (Audenaert et al., 2019b).

Starting from the segmented structures, dense set of correspondences between homologous surfaces in the data set were automatically established by non-rigid mapping of an anthropometric mask (quasi-landmarks) onto the original 3D reconstructions, using a selection of readily available point/surface matching techniques (Audenaert et al., 2019b). Upon completion, all relevant structures in all images were represented as a homologous series of dense landmarks as required for the geometric morphometric analysis and the development of the final statistical analysis of shape of the lower limb anatomy. Note that, by homologous, we mean that each quasi-landmark occupies the same position on each structure relative to all other quasi-landmarks. Following robust Least Squares (Procrustes) superimposition of these homologous series of dense landmarks to account for uninteresting positional, rotational and, possibly, scale differences, the variance/covariance of morphological differences within a body part over a population can be established using Principal Component Analysis (PCA) (Claes et al., 2012a). For each sample a right and a left morphology was available. Both were used in the further modeling pipeline; as they particularly present as coupled data sets with important similarities among bony anatomies, however with relevant and asymmetrical noise in terms of scanning positions.

PCA was originally described by Pearson and later adopted by Fisher and MacKenzie (Jolliffe and Cadima, 2016). In PCA a variable transformation is performed from a set of mutually correlated parameters to a set of principal components (PC) that are orthogonal and therefore uncorrelated. It is one of the oldest and most widely used dimensionality reduction techniques, decomposing a multivariate data set into its mean and corresponding covariance matrix. The eigenvectors of the covariance matrix are usually referred to as PC or eigenmodes, whereas the eigenvalues indicate their relative importance or so-called weight. The first PC is usually called the main mode of variation, as it represents the direction of maximal variance within the data. In the particular case of anatomical data this component nearly always defines size differences between subjects.

PCA was accordingly used to determine the (co-)variance of morphological differences within the data set and a statistical shape model (SSM) (Cootes et al., 1995) was generated, described as

(1) $$S=\bar{S}+\mathrm{Pb}$$

with *S* the shape vector represented as the ordered list of vertex coordinates (following Generalized Procrustes Alignment). $\bar{S}$ defines the corresponding average shape, *P* = (*p*_1_, *p*_2_, …*p*_*t*_) the matrix of eigenvectors of the covariance matrix ${\left(S-\bar{S}\right)}^{T}\left(S-\bar{S}\right)$, and *b* = (*b*_1_, *b*_2_, …*b*_*t*_)^*T*^ a vector of weights.

SSMs of the different articulations (further referred to as articulated SSM) as well as isolated bony structures (further referred to as decoupled SSM) were constructed. Validation of the isolated models has been performed in previous work and covered analysis of accuracy, specificity, generalizability and compactness (Audenaert et al., 2019a).

### 2.2 Regression based learning of neutral limb alignment

We now need to clearly differentiate pose from alignment. We will further use the term ‘*neutral alignment*’ to describe how osteological structures are spatially connected to each other when no movement or loading in any direction is taking place. In contrast, we define ‘*pose*’ as all deviations from the neutral alignment caused by any intended movement or external force applied on the bones and joints. As such, the problem to be solved here can now be defined as how can we learn, based on bony features, how isolated osteological structures are neutrally aligned with each other within the full lower limb?

Considering pose being noise and independent from neutral alignment, we then considered the outcome of a regressed model linking the decoupled anatomies (pose independent) with the articulated shape models (including positional noise) to represent an neutrally aligned shape model (further referred to as regressed SSM) that does not include pose. To do so, a Partial Least Squares (PLS) regression model was built, relating the decoupled anatomies (pose independent) with the articulated models that included pose variation. As predictor values the PC scores of the decoupled anatomies were used, whereas the PC scores of the articulated anatomies were used for response variables. As postural differences between and within subjects (left -right) is unpredictable from the anatomical features of the underlying isolated bony structures, this regressed model (regressed SSM) therefore defined the average neutral alignment of the individual bony entries based on the particular shape features of the bones within the lower limb.

**Fig. 1:**
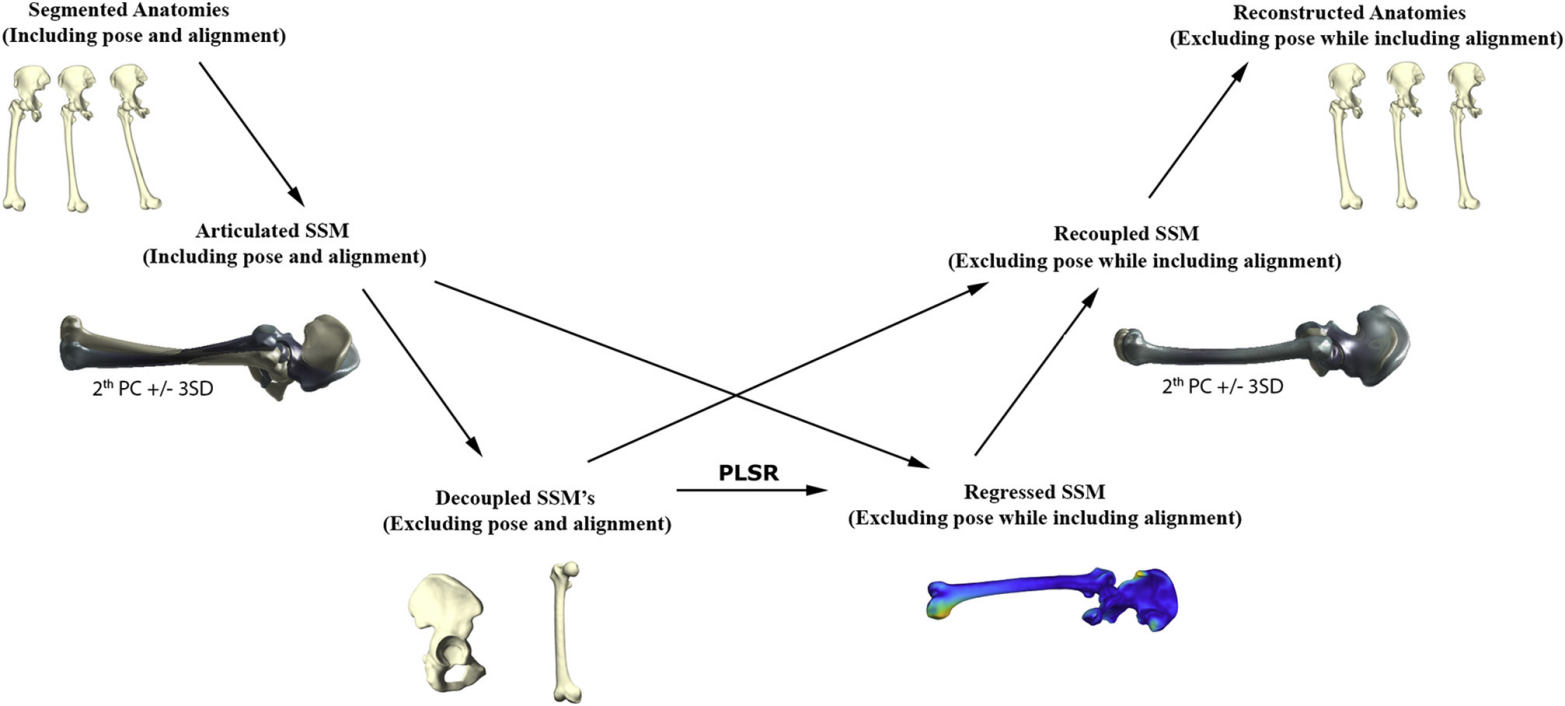
Overview of the general workflow from segmented anatomies to recoupled and reconstructed anatomies.

Finally, as the regressed model defines a -theoretically- neutral aligned and smoothed result, the outcomes of the regressed model were superimposed (Procrustes) with the original decoupled bony anatomies to construct the “recoupled” SSM, in order to preserve all original specifics in anatomical variation. A detailed overview of the workflow of our methodology is presented in Fig. 1.

An error evolution analysis was performed to define the optimal number of PLS regression components to be included in the model. The measure of right/left dissimilarity was assumed to dominantly describe differences in pose between the right and left limb at the time of scanning as opposed to morphological right/left asymmetry. (Audenaert et al., 2019a) The error evolution analysis, when constructing the PLS regression models, was therefore based on the comparison of right/left dissimilarity for an increasing number of PLS components.

### 2.3 Instant prediction of neutral alignment

**Fig. 2:**
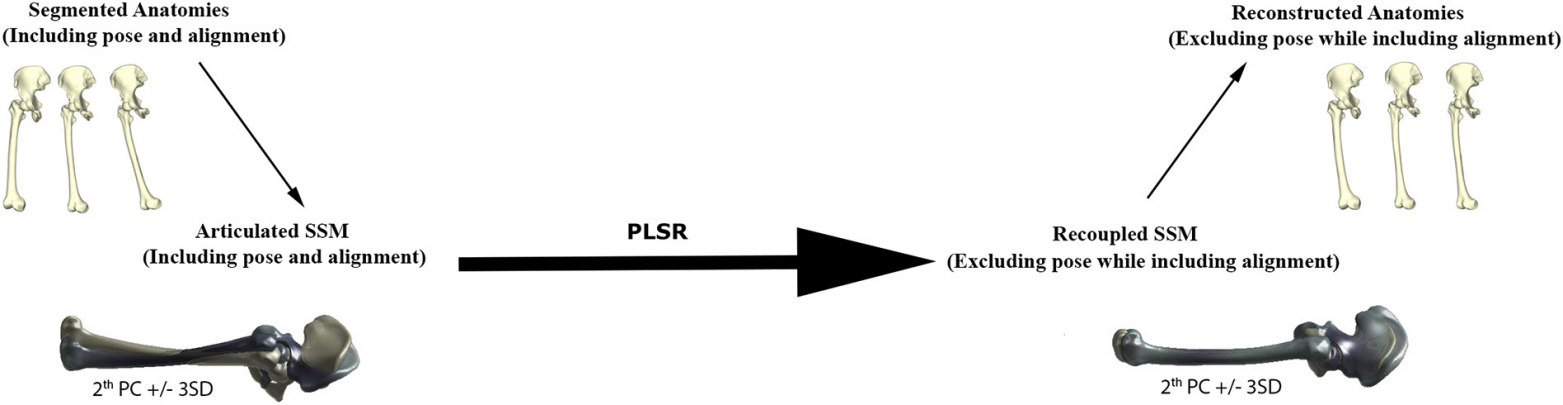
Overview of the workflow from the articulated anatomies to the recoupled and reconstructed anatomies.

For several clinical and research applications, e.g. arthroplasty planning, it would be extremely useful if one could directly predict the neutrally aligned anatomy from the original misaligned or pose corrupted version, without requiring all of the above described steps. To do so, we would need to learn a transformation matrix mapping the articulated data directly to the recoupled data. In essence, learning an additional step, based on the offline processed data from the previous section (Fig. 2). In order to test whether we would indeed be able to instantaneously predict the corrected and neutral aligned anatomies from the articulated anatomies, a second experiment was performed.

Again, a PLS regression model was constructed, this time linking the articulated models (including pose) directly with the recoupled models (excluding pose). In this case the PC scores of the articulated anatomies were defined as predictor values and the PC scores of the previously defined recoupled models as responses. An error evolution analysis was again performed to establish the optimal amount of PLS regression components to be included. The amount of positional noise in the original articulated model was consequently estimated by defining the % of variance in the articulated model that could not be explained by the final recoupled model.

### 2.4 Validation experiments

Two validation experiments were performed. Firstly, validation of the overall methodology was done by predicting the alignment in 80 separate samples (40 right and left combined) whom were scanned repeatedly during the data acquisition period, and as such presented with similar underlying anatomy and neutral alignment, but with differences in pose between scans.

A second validation experiment was based on K = 1000 mock data sets that were create based on the average, neutrally aligned recoupled model shape and its first PC. Random rotations, ranging from −15 to 15° around each principal axis, were applied through idealized joints and the outcomes were registered within the articulated SSM. Following pose denoising using the learned PLS model, the results in term of angular deviations were compared with the original shape entries.

### 2.5 Sexual dimorphism in joint anatomy

PCA of the pose corrected models was performed to describe and analyze variation in joint anatomy in the overall populations as well in comparison between male and female sex. Sexual dimorphism implies sex interactions in patterns of underlying gene expression and function resulting in phenotypic differences between the sexes (Claes et al., 2012b). The sex-shape relationship was evaluated by means of canonical correlation analysis (CCA). In particular, the PC weights of the training data, serving as predictor variables for the male (+1) or female (−1) sex, were used. Overall explained variance in the observed shape components by the factor sex was evaluated by means of partial least squares regression (PLSR). PLSR and CCA are essentially performing the same operation, maximization of sex onto combined shape modes. PLSR and CCA are highly related to each other, however the emphasis is slightly different. In CCA, the aim it to maximize correlation, as it allows for the statistical interpretation of this correlation. In contrast PLSR maximizes the covariance instead of the correlation, and is typically done or used for predictive purposes as a result. In other words, in this work CCA was used to report on the statistical correlation, while PLSR was used to define the predictive value of sex.

## 3 Results

The obtained dataset of training data represented 606 (right and left combined) training samples obtained from 303 imaging data sets, originating from 271 distinct subjects. In this group of subjects, there were 181 male and 90 female subjects, with an average age of 67.8 ( ± 10.8) and 69 ( ± 13.3) years, respectively. During the period of data acquisition, 26 of these persons received secondary (22 subjects) or tertiary CT (2 subjects) scanning for medical reasons, treatment either follow-up. Two patients had a total of 4 scans during the data acquisition period. This allowed us to define 40 couples of rescanned data sets, with matching underlying anatomy, but with differences in pose between scans. These images were included to enrich the model but more importantly for validation purposes.

### 3.1 Regression based learning of neutral limb alignment

**Fig. 3:**
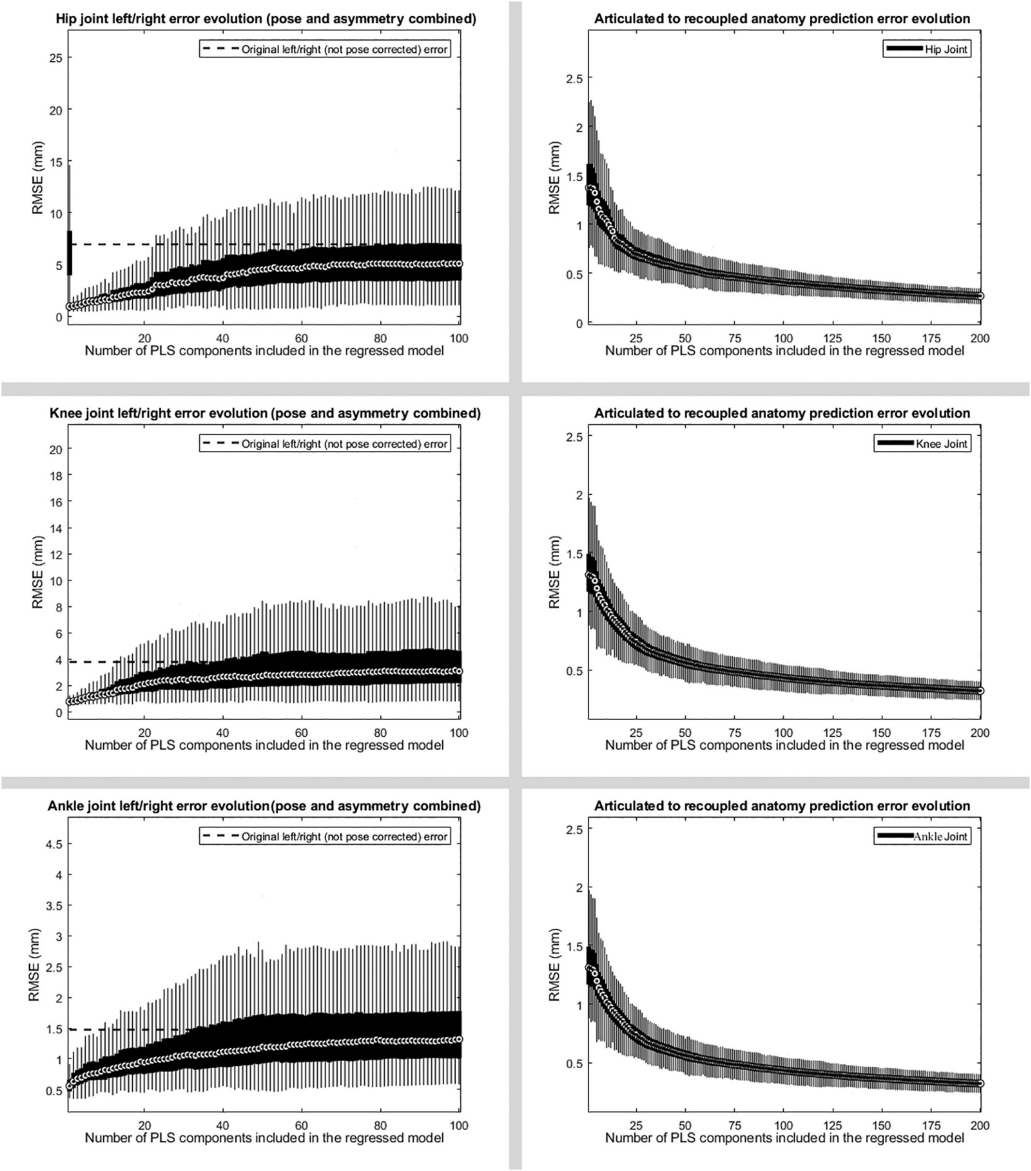
Error evolution (mean left/right dissimilarity) with increasing number of PLS components for the mapping from the decoupled to the regressed models (left) as from the articulated to the recoupled models (right).

The performance of the regressed SSM to reproduce a neutral anatomical alignment in the different lower limb joints was evaluated by comparing left/right asymmetry for which uncorrected pose differences accounted for the majority of inequality in the articulated SSM. When assessing the optimal number of PLS components for the construct of the regressed model it appeared that the vast majority of anatomical variance was explained by the first component (representing 95.13% of explained variance) for the hip. In case of the knee joint 1 component (representing 97.35% of explained variance) demonstrated the smallest reconstruction error. This was as well the case for the first component of the ankle (representing 98.45% of explained variance). Including more components was shown to reintroduce pose variation in the regressed model due to overfitting, demonstrated by a gradual increase in the right/left asymmetry. Details of the analysis are shown in Fig. 3.

### 3.2 Instant prediction of neutral alignment

When learning the direct link from articulated to the recoupled model, however, increasing the number of components did not affect asymmetry findings by the earlier observed reintroduction of pose. Even more, when increasing the number of PLS components up to 200 components, the accuracy of predicting the recoupled anatomies gradually improved up to 0.26 mm ( ± 0.04 mm) in case of the hip joint, 0.23 mm ( ± 0.03 mm) for the knee and 0.21 mm ( ± 0.03 mm) for the ankle joint.

### 3.3 Validation experiments

Model compactness improved strongly following pose neutralization. The first component of the articulated hip model explained only 66.96% of data variance as compared to 83.35% in the recoupled model. In the articulate hip model, pose was estimated to define 8,19% of data variance, as this was the amount of variance in the articulated model which could not be explained by the recoupled model.

For the knee joint, the first component explained only 76.83% of data variance, as compared to 92.77% in the recoupled model. The effect of pose was estimated to account for 6.43% of data variance in the articulated knee model.

The first component of articulated ankle model accounted for 84.67% of data variance while the recoupled first component covered 89.37% of data variance and pose was estimated to account for 2.23% of data variance in the articulated ankle model.

Validation results in terms of extracting positional noise from the articulated model was performed by means of two separate experiments. Firstly, dissimilarity between the rescanned cases, representing unseen and independent articulated variations, demonstrated an accuracy of 0.03 to 0.08° or 0.21 mm to 0.26 mm. Similar results were obtained in the mock dataset on which virtual pose differences were imposed. A detailed overview of these validation results is presented in Table 1.

**Table 1:** Validation results on the mock and rescanned cases, representing unseen and independent articulated variations.

		Virtual cases (n = 1000)			Rescanned cases (n = 80)		
		Difference with control before pose correction	Difference with control after pose correction	p	Within-patient difference before pose correction	Within-patient difference after pose correction	p
Hip	Flexion (degrees)	7,64 ± 4,26°	0,13 ± 0,08°	<0,001	1,74 ± 1,2°	0,05 ± 0,03°	<0,001
	Abduction (degrees)	7,54 ± 4,01°	0,18 ± 0,13°	<0,001	2,31 ± 1,58°	0,04 ± 0,03°	<0,001
	Endorotation (degrees)	7,46 ± 4,26°	0,26 ± 0,18°	<0,001	5,58 ± 3,62°	0,08 ± 0,09°	<0,001
	Distance (RMSE)	13,07 ± 5,92 mm	0,48 ± 0,23 mm	<0,001	5,14 ± 3,08mm	0,26 ± 0,11mm	<0,001
Knee	Flexion (degrees)	7,48 ± 4,18°	0,21 ± 0,14°	<0,001	1,24 ± 1,2°	0,03 ± 0,03°	<0,001
	Abduction (degrees)	7,52 ± 4,21°	0,16+/- 0,13°	<0,001	0,61 ± 0,66°	0,006 ± 0,006°	<0,001
	Endorotation (degrees)	7,46 ± 4,35°	0,31 ± 0,21°	<0,001	1,06 ± 0,99°	0,001 ± 0,002°	<0,001
	Distance (RMSE)	21,76 ± 9,18 mm	0,74 ± 0,43 mm	<0,001	2,75 ± 1,69 mm	0,21 ± 0,08 mm	<0,001
Ankle	Flexion (degrees)	7,68 ± 4,32°	0,13 ± 0,06°	<0,001	2,61 ± 2,68°	0,02 ± 0,02°	<0,001
	Abduction (degrees)	7,53 ± 4,24°	0,15 ± 0,07°	<0,001	2,27 ± 2,61°	0,01 ± 0,01°	<0,001
	Endorotation (degrees)	7,47 ± 4,29°	0,14 ± 0,09°	<0,001	2,12 ± 2,05°	0,01 ± 0,01°	<0,001
	Distance (RMSE)	4,32 ± 2,16 mm	0,28 ± 0,08 mm	<0,001	1,81 ± 0,74 mm	0,24 ± 0,16mm	<0,001

### 3.4 Sex dimorphism

**Fig. 4:**
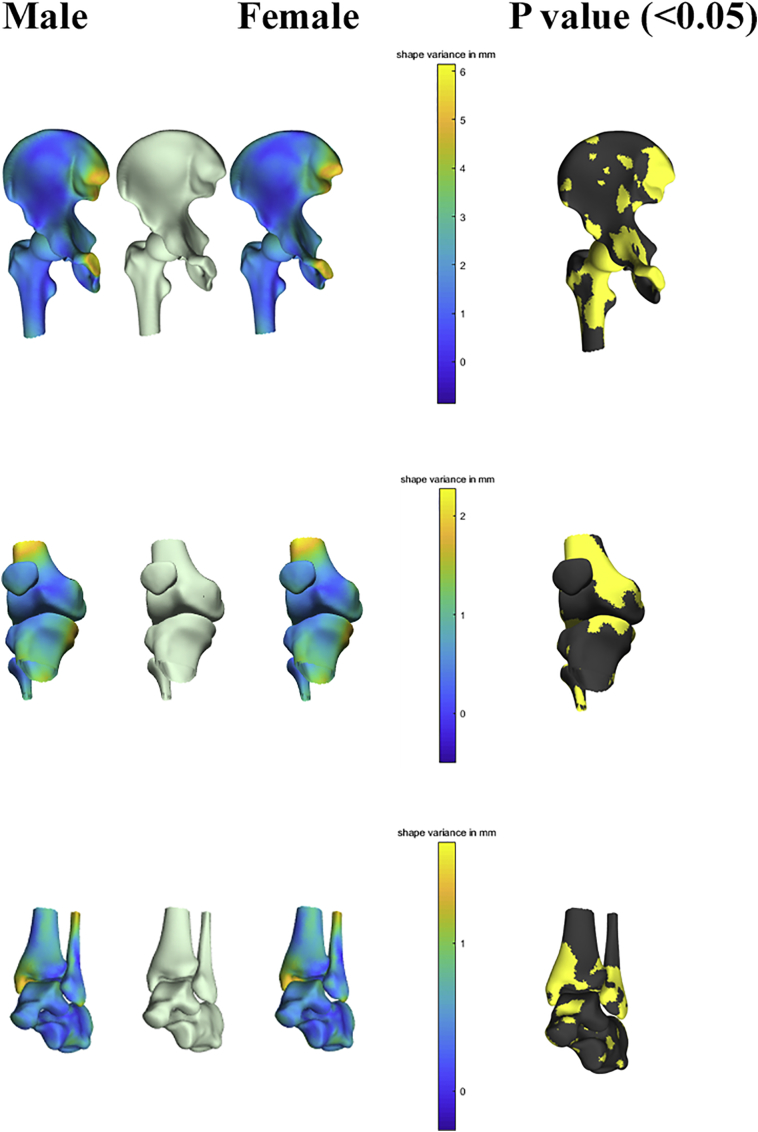
Color mapping of the observed Male/female differences in joint anatomy as obtained following the canonical correlation. Average male/female canonical scores were amplified with a factor 2 and corrected for overall size to visualize the differences between the obtained surfaces (left). Statistical significance of the findings was mapped on the average model right).

Sex accounted for 8.53% of anatomical variance of the hip (*r* = 0.95; *p* < 0.001), 29.19% of variance around the knee (*r* = 0.91; *p* < 0.001) and 38.53% of variance about the ankle (*r* = 0.89; *p* < 0.001). Size was most pronounced present in the first PC and this was as well the most sex discriminative PC for all joints. Other distinguishable features, relating more implicitly to shape as compared to size, include narrower subpubic angle and overall narrower pelvic inlet in males as compared to females. For the femur, clinically relevant particularities were as well observed. Although female subjects have overall smaller sizes, a decreased cortical radius and a relatively smaller diameters of the femoral head was noticed. Around the knee, most noticeable features were increased condylar width and size of the tibia plateau in male samples. Distinct difference between male and female around the ankle related to the width of ankle mortise and the length of the fibula tip, which was larger in male subjects. Male/female differences as obtained following the canonical correlation analysis are demonstrated in Fig. 4. Average male/female canonical scores were amplified with a factor 2 and corrected for overall size.

**Fig. 5:**
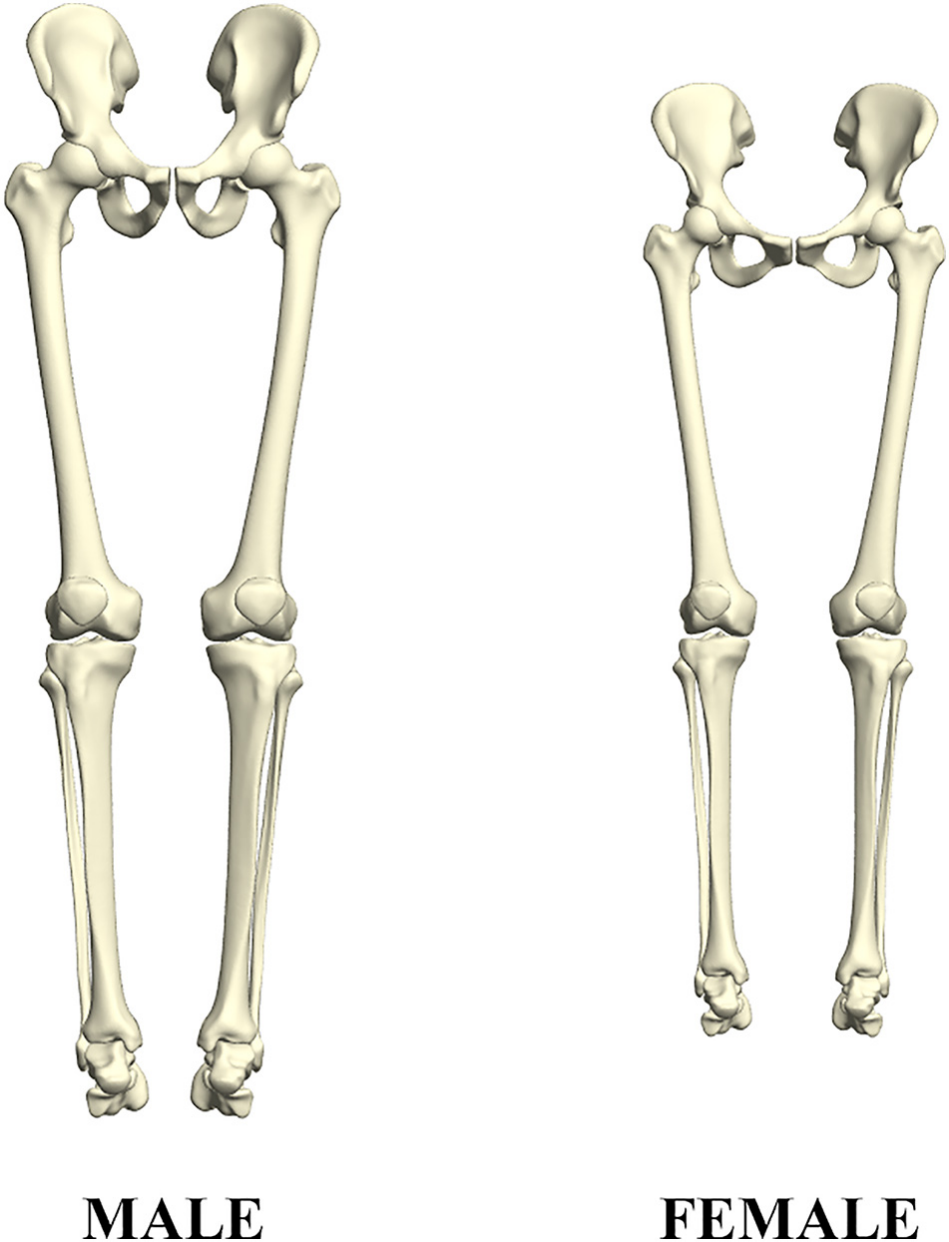
Male versus female whole lower limb anatomy as obtained following canonical correlation analysis. Average male/female canonical scores were amplified with a factor 2.

An illustration of the overall difference in male versus female morphology of the whole lower limb in neutral alignment is presented in Fig. 5. Reconstruction of the full lower limb was performed by consecutively correcting for pose in the ankle, knee and the hip joint. Again, for visualization purposes, average male/female canonical scores were amplified with a factor 2. It was found that sex accounted for as much as 38.9% (*r* = 0.94, *p* < 0.001) of anatomical variance in the recoupled model, as compared to 37.8% (*r* = 0.92, *p* < 0.001) in the articulated model (thus including pose), and taking into account the first 20 PCs.

### 3.5 Impact of noise reduction on SSM performance

**Fig. 6:**
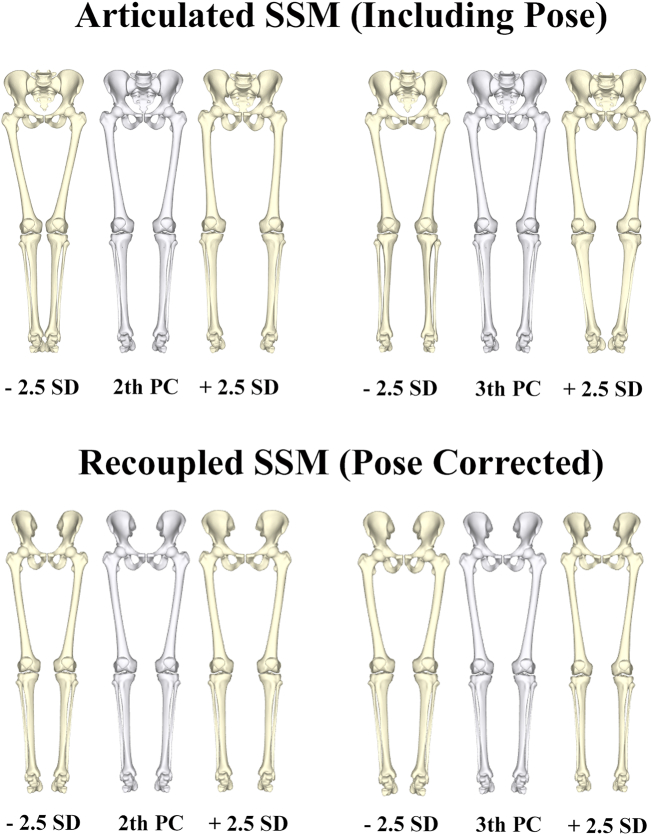
Although the first PC in both models was very comparable and related mainly to difference in size between subject, there were pronounced differences in the 2th and 3th PCs between the two models. In particular, in the articulated model pose was dominant on the major PCs, with the second PC describing closed versus open leg positions and the 3th PC rotations of the lower limb.

Separating the underlying anatomy from variation induced by difference in pose had important impact on a number of qualitative measures of the model. While the main variation in both models (first PC) was caused by size difference between subjects, the recoupled model was found to be significantly more compact as compared to the original articulated model. This is mainly due to the fact that the first PC modes of the articulated model picked up important covariance caused by purely positional and rotational differences in the data. Fig. 6 demonstrates the 2th and 3th PC of the articulated model in comparison to the recoupled model.

**Fig. 7:**
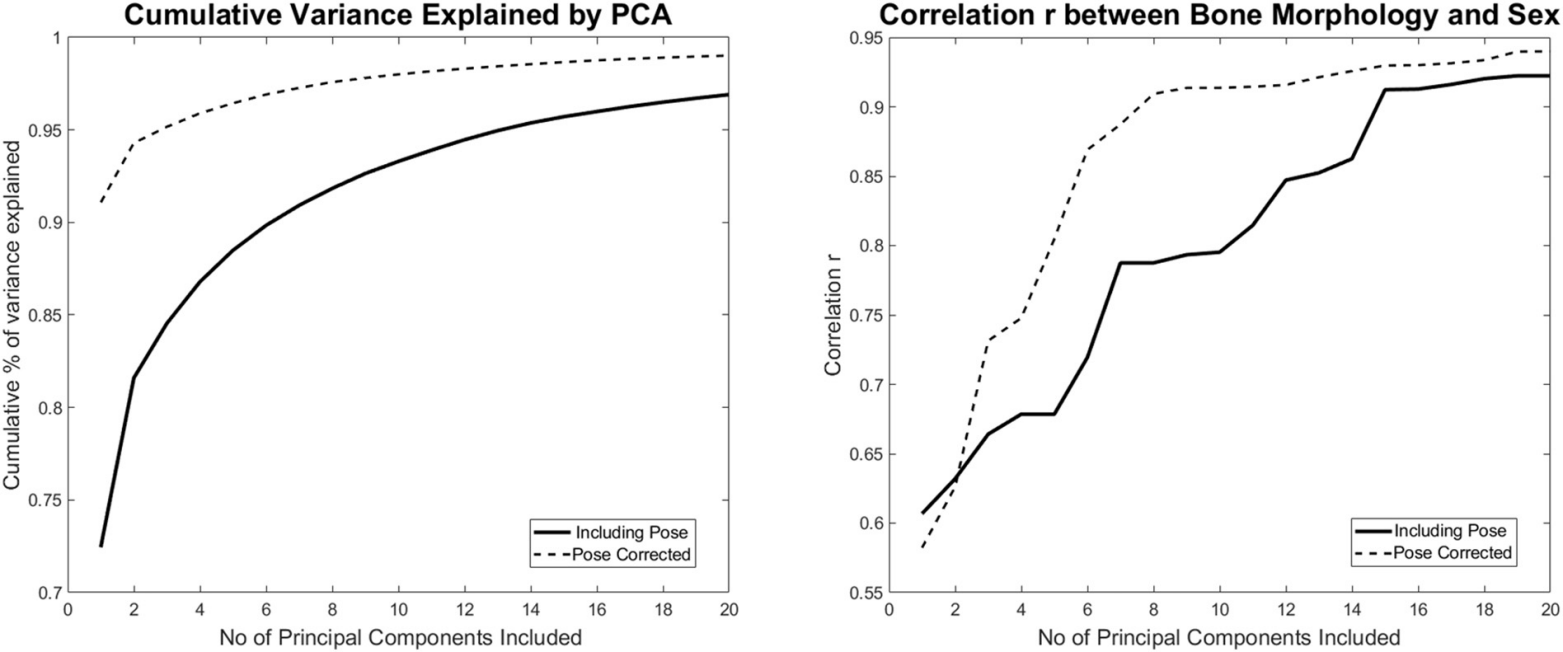
Cumulative variance of the population by number principal components following a principal component analysis (PCA) to describe the articulated and recoupled model of the full lower limb. (Left) Canonical correlation analysis results relating variation in shape with for the articulated (including pose) and recoupled model (pose corrected) (right).

The 2th PC of the articulated models demonstrated closed legs versus open legs in the articulated (pose including) model (Abduction and adduction around the hip). Similarly, the 3th PC of the articulated model presented major positional interference, mainly internal versus external rotation of the limb. In contrast, no apparent pose related variation was noticed in the recoupled model. In the recoupled model, the 2th PC mainly demonstrated variance in width of the pelvis and related difference in the size of the support base, whereas the 3th PC demonstrated differences in the radius of the long bones mainly. These advantages of noise reduction in the recoupled model became further apparent in the shape versus sex correlation analysis, plateauing already at 8 components in the recoupled model whereas the articulated model required at least double to amount of components. An overview of the differences between both models in terms of compactness and sex correlation with increasing number of PCs taken into account is presented in Fig. 7.

## 4 Discussion

In the present manuscript we present a first in its kind methodology to separate positional noise from neutral joint alignment. The performed validation experiments demonstrate that we achieved this within submillimeter and below 1-degree prediction error range. Further, it was demonstrated that the neutral alignment model was by far superior in terms of compactness (computational efficiency) and its capability of associating predictive variables such as sex. Articulated shape models that were presented in the past, have been explicitly developed for use in segmentation task, in which positional variation in the data is actually aimed for. In research areas like anatomical, forensics, computational genetics and evolutionary biology, however, positional information is considered noise as it adds unwanted variance in the data and obscures in depth analysis of correlation between anatomy of joints and other variables at interest.

Over the last years, there has been a growing interest in genetic association studies with shape. Already several single-nucleotide polymorphisms (SNP) have been identified independently associated with bone morphology (Baird et al., 2019; Lindner et al., 2015). These studies are being performed to increase the understanding of risk and predictor factors for osteoarthritis and fracture. Al of these have been performed on 2D image databases. We therefore believe that the present work can add significantly to this research domain as it will allow for smaller data requirements while providing enhanced accuracy in the anatomical definitions of shape, by excluding positional noise.

Joint alignment has been consistently identified as an independent and crucial risk factor for the development of early degeneration of the joints (Hunter et al., 2005; Vandekerckhove et al., 2017). Patients presenting with clinical complaints and early stage radiographical abnormalities are frequent as are the conservative and surgical means to deal with them. Given the socioeconomic burden of aging and in particular joint replacement surgery, disease modifying interventions have never been more popular ranging from bracing – e.g.; “unloaders” to mechanical implants (e.g. Kine-Spring®) and surgical corrections such as varisation/valgisation osteotomies. However, when it comes down to clinical decision making it remains largely unsolved whether a patients conditions is constitutional either progressive degenerative and whether the condition is amendable by physiotherapy or soft procedures, or whether correction of the neutral anatomy in terms of osteotomy is the preferable way to restore the mechanical joint environment (Vandekerckhove et al., 2017). Furthermore, when finally deciding on joint replacement surgery, recent studies have suggested superior outcome in constitutional alignment restoration as compared to neutral mechanical alignment upon TKP placement (Liau et al., 2002; Magnussen et al., 2011; Matziolis et al., 2010; Miller et al., 2014; Vanlommel et al., 2013). Besides these clinical observations, mechanical studies have additionally demonstrated that restoration of constitutional alignment restores physiological strain in periarticular tissues and ligaments (Delport et al., 2015). With the technique presented, a fast methodology becomes available to analyze a person's native and natural, constitutional alignment and to describe in what way and how much it deviates from the average. Besides an improved diagnostics, the presented methodology can also aid in optimizing treatment planning for both osteotomy and joint replacement surgery by restoration of the alignment.

A secondary aim of this study was to evaluate sex differences in joint anatomy. In particular, the pelvis, given its obstetric relation, has been the focus in the past. Studies of sexual dimorphism in the human pelvis show that while, in general, many pelvic characteristics reflect full body size, and are therefore larger in men than in women, other dimensions of the pelvic canal follow the inverse model (Kurki, 2011; Tague, 2000). Our findings also seem to support some relevant clinical findings around the knee where sex differences have been a recent subject of interest in the area of knee arthroplasty (Thomsen et al., 2012). A significant difference in knee width was demonstrated between male and female samples and presented the most pronounced component of variation (excluding size). This is in agreement with previous clinical reports (Chin et al., 2002; Hitt et al., 2003) and the industry has even adopted this concept for the development of gender specific implants (Thomsen et al., 2012). Although interesting from a commercial point of view, no evidence of any outcome advantage in the gender-specific design, however, has been demonstrated in randomized clinical trials (Thomsen et al., 2012).

When evaluating the overall difference in male versus female phenotype, the strongest differences were found in size, cortical thickness, pelvic width and related size of the support base. All of the above have important mechanical consequences and might therefore be associated with occurrence and gender specific prevalence of some common musculoskeletal pathologies including gluteal tendinitis (increased moment in females), knee (increased adduction moment in females) and ankle osteoarthrosis. Other authors, based on anthropometric measures, have similarly attributed a possible superior postural stability in females as compared to males (Sell et al., 2018). According to Hayes et al., the stability of the individual is inversely proportional to the center of gravity height, and directly proportional to the support base, and these variables are related with postural balance and more optimal in a female phenotype (Hayes, 1982). Further studies in sex differences in biomechanics, anatomy and related pathology are definitely warranted.

An import limitation of our findings relates to the population that was investigated, which is a single homogenous population of European Descent, namely Belgian people. It is unknown to what extent our findings can be extrapolated to other populations. The complex interaction between genes, culture and the environment results in a population-based variation, with several studies showing that the appropriate evaluation of this variation requires specific standards for each population (Rissech et al., 2013; San-Millan et al., 2017). Nevertheless, in general terms we expect our results to be representative by extension for a Western European population.

A second limitation refers to potential selection bias and sample size. We previously established estimates of sample size required for anatomical studies on human anatomy and found 200 samples to be sufficient for our data type to be population covering (Audenaert et al., 2019c). Conversely, these numbers can further increase for heterogeneous and admixed populations. In terms of selection bias, it needs to be mentioned that people with arthritic joints were excluded from analysis. Further studies on adolescents and young adults seem mandatory in the future the refine our findings.

Finally, it would have been valuable to investigate and remove the effect of height and weight differences on the analysis presented. We did not have, however, this potentially interfering information within the cohort.

In conclusion, the current manuscript presents an accurate technique for denoising positional variance in anatomical data that can be generically applied to any joint. Further, once the models are trained, these tools can be implemented in real time on registered data sets.

## Funding

Emmanuel Audenaert was financially supported by a senior clinical research fellowship from the Research Foundation Flanders.

## Declaration of competing interest

The authors declare that they have no competing interests that could inappropriately influence this work.
